# Supplementary material for: Cross-species evaluation of TANGO2 homologs, including HRG-9 and HRG-10 in Caenorhabditis elegans, challenges a proposed role in heme trafficking
Source: eLife. 2026 Jan 8;14:RP105418. doi: 10.7554/eLife.105418 (PMC12782551; doi:10.7554/eLife.105418)
Supplement: Figure 4—source data 1. [file elife-105418-fig4-data1.zip › Figure 4-source data 1.pdf]

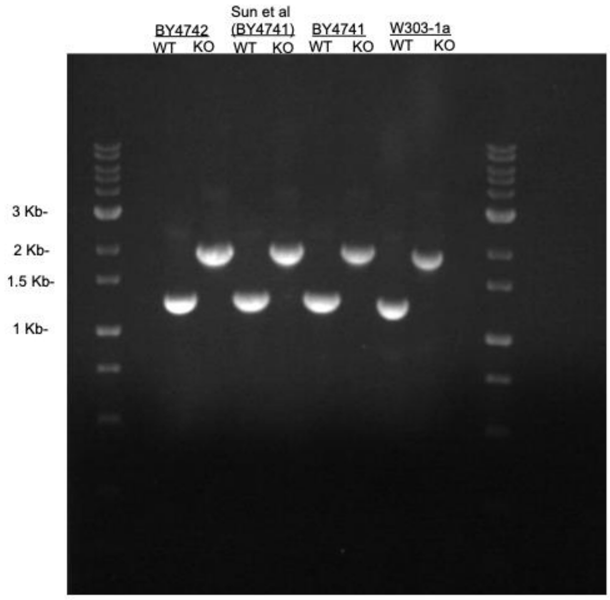


**Source data 2** – The uncropped gel with the relevant bands clearly labelled, corresponding to panel A of Figure 4.
